# The elongation factor eEF3 (Yef3) interacts with mRNA in a translation independent manner

**DOI:** 10.1186/s12867-015-0045-5

**Published:** 2015-09-24

**Authors:** Nitzan Samra, Avigail Atir-Lande, Lilach Pnueli, Yoav Arava

**Affiliations:** Department of Biology, Technion-Israel Institute of Technology, 32000 Haifa, Israel

**Keywords:** RNA-binding proteins, Elongation factor 3, Yef3, 3′ UTR, Yeast, Protein translation

## Abstract

**Background:**

mRNA binding proteins (RBPs) constitute 10–15 % of the eukaryotic proteome and play important part in post-transcriptional regulation of gene expression. Due to the instability of RNA and the transient nature its interaction with RBPs, identification of novel RBPs is a significant challenge. Recently, a novel methodology for RBP purification and identification (termed RaPID) was presented, which allows high affinity purification of RBPs while associated with mRNA in vivo.

**Results:**

We performed a RaPID screen for proteins that interact with PMP1 mRNA in order to identify novel mRNA binding proteins. PMP1 mRNA was tagged in its 3′ UTR with multiple MS2 loops and co-expressed with MS2-binding protein fused to streptavidin binding protein (SBP). RNA–protein complexes were cross-linked in vivo and isolated through streptavidin beads. The eluted proteins were subjected to mass spectroscopy analysis. The screen identified many proteins, about half of them were previously shown to bind RNA. We focused on eEF3 (YEF3), an essential translation elongation factor that interacts with ribosomes. Purification of TAP-tagged Yef3 with its associated RNAs confirmed that the native PMP1 transcript is associated with it. Intriguingly, high association with Yef3-TAP was observed when purification was performed in the presence of EDTA, and with PMP1 that contains stop codons immediately downstream to the initiation codon. Furthermore, high association was observed with a transcript containing only the 3′ UTR of PMP1. Complementary, RaPID isolation of MS2-tagged 3′ UTRs with their associated proteins revealed that Yef3 can efficiently interact with these regions.

**Conclusions:**

This study identifies many novel proteins that interact with PMP1 mRNA. Importantly, the elongation factor Yef3 was found to interact with mRNA in non-coding regions and in a translation independent manner. These results suggest an additional, non-elongation function for this factor.

**Electronic supplementary material:**

The online version of this article (doi:10.1186/s12867-015-0045-5) contains supplementary material, which is available to authorized users.

## Background

RNA binding proteins (RBPs) represent about 10 % of the total proteins in the cell in *S. cerevisiae* [1–3] and about 15 % in mammals [4]. They are implicated in many cellular processes including mRNA post-transcriptional processing and regulation, translation, ribosomes biogenesis, tRNA aminoacylation and modification, chromatin remodeling and more. Furthermore, some RBPs function together with RNA molecules in ribonucleoprotein complexes (RNPs) to perform distinct functions. The most known examples are ribosome in translation, telomerase in DNA’s end elongation, splicosome in pre-mRNA processing and the signal recognition particle (SRP) in cellular targeting. An important subgroup of RBPs is the mRNA binding proteins (mRNPs). In the course of mRNA’s maturation, different RBPs bind the transcript and mediate its nuclear processing, export out of the nucleus, cellular localization, translation and degradation. The distinct set of RBPs bound to an mRNA molecule at any time point determines how it will be processed and ultimately its fate [5, 6]. mRNAs are therefore likely to be associated with many proteins throughout their life time. To date, however, the repertoire of proteins that is associated with a particular mRNA is not known.

In recent years, many novel RBPs were identified. Intriguingly, many of these proteins were known to be executing other cellular functions (such functions are referred to as moonlighting functions) [7]. In particular, metabolic enzymes were found to bind mRNAs, thereby suggesting coordination between the cellular metabolic state and post-transcriptional regulation [2–4].

In addition to novel RBPs, recent studies had identified novel regulatory functions to known RBP. Many ribosomal proteins were found to have extra-ribosomal functions, including auto-regulation of their expression [7–10]. They were shown to interact with non-coding RNAs [11] or non-coding domains [12] and provide a regulatory role. Regulation can be through any aspect of mRNA expression, including translation regulation, mRNA stability or splicing [13–16].

Translation elongation entails the function of several elongation factors. These are either involved in tRNA binding and targeting it to the ribosome, or bind the ribosome and assist in its translocation along the coding region. Interestingly, the eukaryotic elongation factor 1A (eEF1A) was shown to have many roles beyond its role in tRNA binding. It was found to also bind sequence in the 3′ UTR of the MT-1 mRNA, which is important for mRNA localization [17, 18]. Furthermore, it can directly bind actin mRNAs and affect their localization to cellular protrusions [19]. EF1A is also known to bind non-coding RNAs such as HSR1 and tRNAs [20]. Recently, one of EF1A isoforms was shown to interact with HSP70 mRNA and post-transcriptionally regulate its levels [21]. These studies demonstrate that elongation factors can confer different roles by binding different types of RNAs and at different stages of the gene expression program.

In this study, we screened for novel proteins that interact with the *S. cerevisiae* PMP1 mRNA. PMP1 encodes a small proteolipid that is associated with the plasma membrane H (+) ATPase and enhance its activity [22]. PMP1 mRNA was shown to be associated with ER in a manner that depends on its 3′ UTR [23]. The proteins that are important for this association are not known [24]. RaPID analysis in yeast cells revealed proteins that interact with the mRNA, most of them are RNA binding proteins. We followed up on the elongation factor Yef3 (eEF3) and confirmed its association with the native PMP1 by utilizing TAP-tagged protein. Furthermore, we show that Yef3 interacts with mRNA in a ribosome-independent manner, and can bind 3′ UTRs. These results suggest a novel regulatory role for eEF3.

## Results

### Establishing RaPID for PMP1 3′ UTR

**Fig. 1:**
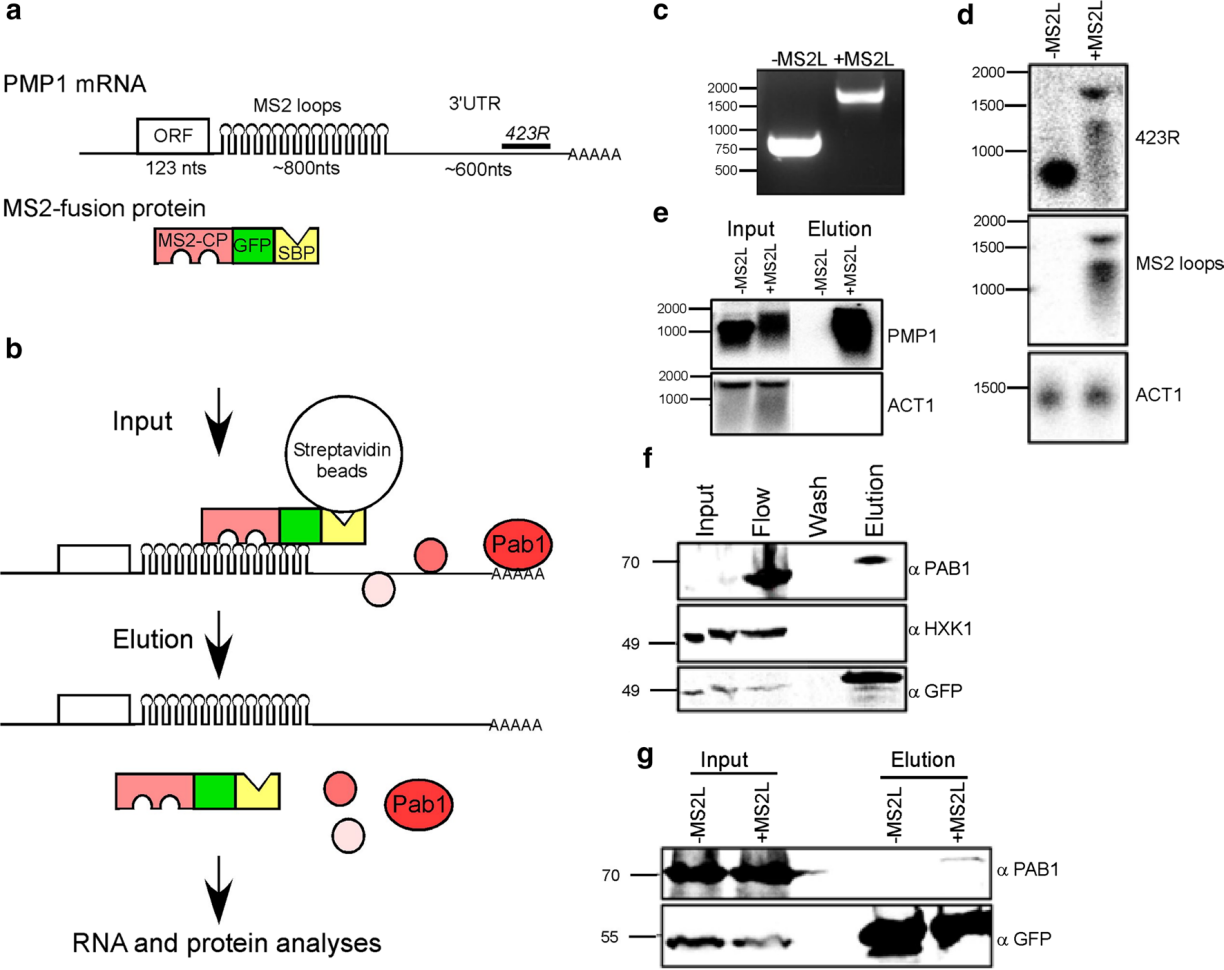
Purification of PMP1-assocaited proteins by RaPID. **a** Scheme of the tagged PMP1 mRNA and fusion MS2 binding protein. Lengths of the different mRNA regions are indicated. *423R* indicates the position of the antisense oligo probe that was used in D (complementary to a region 380–423 nts downstream to the stop codon). The MS2 fusion protein includes an MS2 binding domain (MS2-CP, *pink*) fused in frame with GFP (*green*) and Streptavidin binding protein (SBP, *yellow*). **b** Key steps in RaPID. Protein lysate from cells expressing the tagged PMP1 and the MS2 fusion protein (*input*) is loaded on Streptavidin beads, subjected to extensive washes and bound material is eluted by addition of biotin. The eluted material is subjected to RNA analysis (*panels* **d** and **e**) or protein analysis (**f**, **g**). Ultimately, protein samples are subjected to mass spectroscopy to identify novel RNA binding proteins depicted as red circles. **c** PCR analysis for genomic DNA that was extracted either from cells with normal PMP1 (−MS2L) or cells that were subjected to MS2 loops insertion (+MS2L). PCR primers are from PMP1 promoter (forward) and 3′ UTR (reverse). DNA size markers are indicated to the *left*. **d** RNA was extracted from the indicated strains by the hot phenol method, and subjected to northern analysis with the indicated probes. RNA size markers are indicated to the *left*. **e** The indicated strains were subjected to RaPID, and RNA samples extracted from 5 % of the input lysate (*input*) or from half of the eluted material (*elution*) were subjected to northern analysis with the indicated probes. **f** Protein samples were collected along the RaPID protocol, and subjected to western analysis with the indicated antibodies. Samples from the following steps were analyzed: Input lysate (*input*), flow through (*flow*), last wash (*wash*) and eluted material (*elution*). Protein size markers are indicated to the *left*. Note that the Input lane seems split due to electrophoresis problem. **g** RaPID analysis was performed to cells either expressing untagged PMP1 (−MS2L) or tagged PMP1 (+MS2L). Protein samples from the Input or the Elution were subjected to western analysis with the indicated antibodies

We utilized the RaPID method to identify novel proteins that interact with PMP1 mRNA in *S.cerevisiae* [25]. In this method, the target mRNA is tagged by multiple MS2 loops, which serve as a binding site for the MS2 coat protein (MS2-CP). MS2-CP is co expressed as a fusion with GFP (for fluorescent visualization) and streptavidin binding domain (SBP) (Fig. 1b). Thus, a cell lysate (Input) can be loaded on streptavidin conjugated beads, non-specific binders can be washed away (owing to the specific and strong binding of MS2-CP to the MS2 loops and of SBP to the streptavidin beads). Novel RNA-binding proteins can be identified by mass spectroscopy from material eluted from the beads (Fig. 1a). To enable *PMP1* mRNA isolation, a sequence consisted of 12 MS2 binding sites (MS2 loops; MS2L) was inserted to the genomic locus of *PMP1* between the ORF and 3′ UTR by homologous recombination (in the following genetic background BY4742 Mat α, *his3∆1*, *leu2∆0*, *lys2∆0*, *ura3∆0*) (Fig. 1a) [26]. A PCR reaction with primers from outside of the expected MS2 loops insertion site showed a single product about 800 nts longer than the untagged *PMP1* fragment (Fig. 1c) indicating that the MS2 loops were fully inserted into *PMP1* gene.

In order to verify that the MS2 loops insertion did not alter PMP1 3′ UTR expression, RNA was extracted from cells expressing *PMP1* with or without MS2 loops, followed by northern analysis. A DNA oligonucleotide corresponding to 381-423nts downstream to *PMP1* stop codon was radioactively labeled and used as a probe (Fig. 1d, 423R probe). *PMP1* without MS2 loops show a strong signal at a size of ~900 nts, corresponding to the expected size of *PMP1* mRNA [23]. *PMP1* with MS2 loops (*PMP1*-*MS2L*) shows a signal ~1700 nts long, which is the expected size of *PMP1* with the addition of the MS2 loops (~800 nts). This signal appears weaker than the untagged *PMP1* mRNA, suggesting an impact on *PMP1*-*MS2L* stability. Consistent with that, a weaker and less distinct band appears below 1500 nts, most likely a degradation product of the full length transcript. The two bands were also detected with a probe corresponding to the MS2 loops sequence (Fig. 1d, MS2 probe), confirming that the MS2 loops exist in both transcripts. A probe corresponding to the ORF of actin (*ACT1*) was used to confirm that similar amount of RNA were loaded in all lanes. Taken together, these results show that a full length transcript of ~1700 nts of *PMP1*-*MS2L* is expressed.

A common drawback of purification protocols is the presence of non-specific binding. To evaluate RaPID ability to specifically isolate *PMP1* mRNA, yeast strains expressing *PMP1* with or without MS2 loops were transformed with a plasmid expressing MS2-CP-GFP-SBP fusion protein. Cells were grown to mid log phase, transferred to induction medium and then fixed with formaldehyde. Cells were than harvested and lysed and the lysate was incubated with streptavidin beads. The beads were then washed and bound material was eluted and tested for specific mRNAs by northern analysis. As can be seen in Fig. 1e, neither the untagged *PMP1* mRNA, nor a control ACT1 mRNA precipitates with the streptavidin beads. Only the MS2L-tagged transcript precipitates with the streptavidin beads. These results confirm the specific isolation of *PMP1* through the MS2 loops, without nonspecific binding of other mRNAs to the streptavidin beads or the MS2-CP-GFP-SBP fusion protein.

To further establish the RaPID procedure, we tested for proteins that are known to bind mRNA. Yeast cells expressing *PMP1*-*MS2L* tagged mRNA and MS2-CP-GFP-SBP fusion proteins were grown in selective medium and were subjected to RaPID procedure. Proteins samples were analyzed by Western blot. As can be seen in Fig. 1f the polyA binding protein Pab1p is efficiently precipitated with the beads, while the control protein HXK1 (which is not known to bind PMP1) does not. Pab1 precipitation is apparent when PMP1 is tagged with MS2L and does not occur when PMP1 is not tagged (Fig. 1g). Overall, these results indicate that the RaPID method successfully isolate specific proteins that are associated with *PMP1*-*MS2L* mRNA.

### Identification of novel proteins that associate with *PMP1* mRNA

**Fig. 2:**
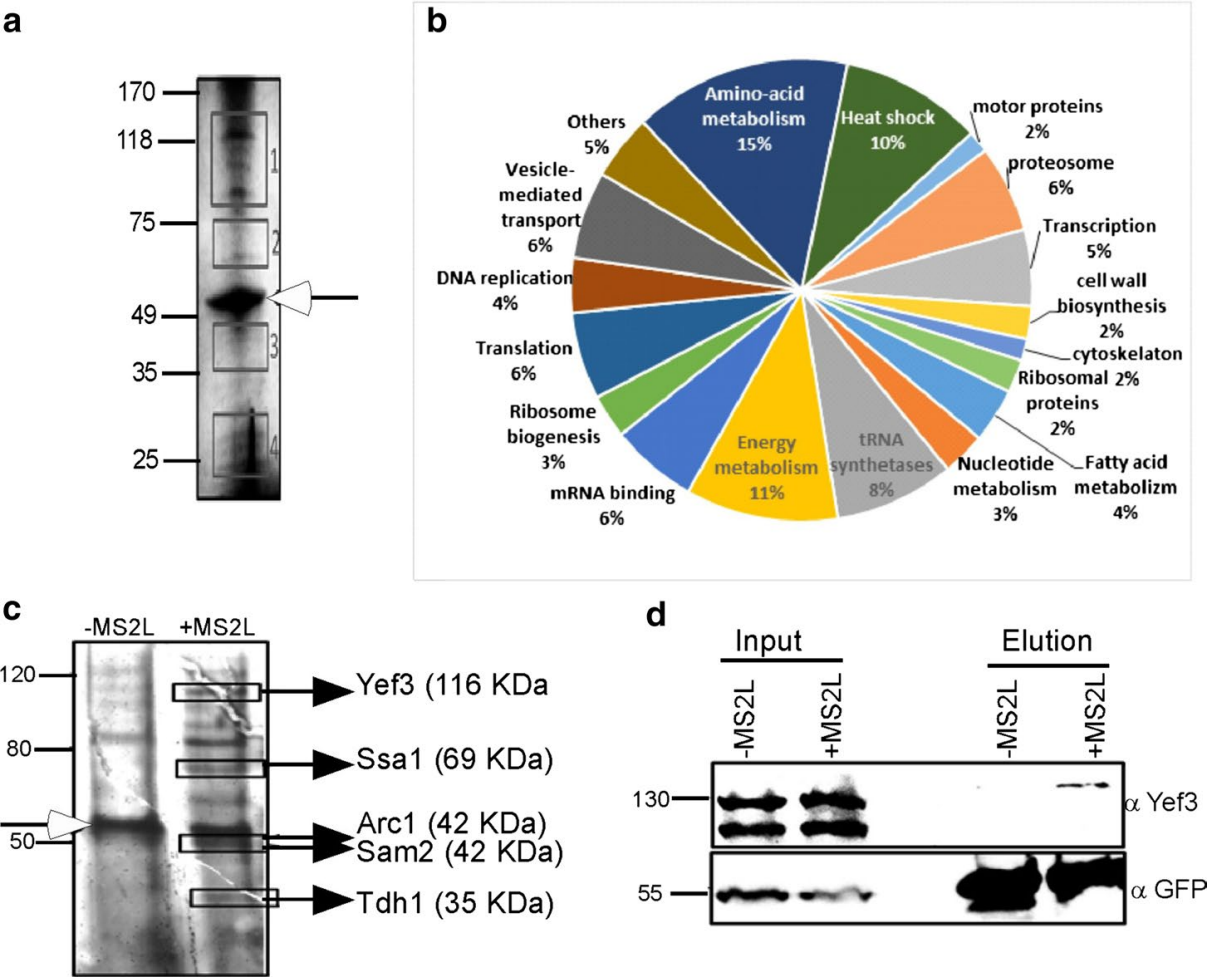
Mass Spectroscopy analyses for novel RBPs: **a** Protein elute from RaPID was subjected to PAGE electrophoresis and silver-stained. The areas marked by *squares* were excised from the gel and subjected to mass spectroscopy. Protein size markers (in kDa) are indicated to the *left*. *Open arrow* indicates the MS2 fusion protein. **b** The proteins with significant signals in the mass spec. analysis (Additional file 1: Table S1) were grouped according to their function. **c** RaPID was performed to cells with either untagged PMP1 (−MS2L) or tagged mRNA (+MS2L). Protein samples were resolved on PAGE, silver stained, and bands that appeared stronger in the +MS2L (marked by *squares*) were excised and subjected to mass spec. Protein size markers (in kDa) are indicated to the *left.* *Open arrow* indicate the band corresponding in size to MS2 fusion protein. **d** Western analysis confirmation for Yef3 association with PMP1. RaPID was performed to the indicated strains and samples from the Input and the Elution were subjected to western analysis with the indicated antibodies. Note that Yef3 is usually detected as two bands due to cleavage [31]

Very few proteins are known to bind *PMP1* mRNA [24] and none of them appeared to be involved in its localization. We therefore wished to characterize the *PMP1* mRNA-protein complex (mRNP) by finding yet unknown proteins associated with it. For this purpose, RaPID procedure was performed, proteins from the eluted material were separated on polyacrylamide gel and subjected to silver staining. Several bands are visible including a prominent MS2-CP-GFP-SBP fusion protein (Fig. 2a). In order to identify those proteins, four areas containing visible bands were cut from the gel, extracted, digested with trypsin and analyzed by liquid chromatography tandem mass spectrometry (LC–MS/MS). 134 proteins were identified (Additional file 1: Table S1) and manually classified into 19 categories (Fig. 2b).

We also utilized the GO term analysis tool from SGD, to search for protein families that are statistically enriched. Proteins were analyzed according to their cellular function (F), the process they are involved in (P) or the complex that they are part of (C) (Table 1). A significant enrichment was observed to functions and complexes that are related to RNA metabolism, including mRNA binding, tRNA synthetases, ribonucleoprotein granules or cytoplasmic granules. This significant enrichment indicates that RNA associated proteins are specifically enriched by the RaPID pull down assay.

**Table 1:** GO term analysis of Mass Spec. results

Gene names	*P* value	Cluster frequency (%)	Background frequency (%)	GO term	GO id
ILS1, KRS1, YDR341C, GUS1, VAS1, MES1, YHR020W, THS1, DPS1, CDC60	5.74E−08	7.80	0.50	Aminoacyl-tRNA ligase activity (F)	4812
DHH1, KRS1, SUP35, PAB1, XRN1, GUS1, TIF4631, SCP160, NAB6, CLU1, NAM7, PFK2, STI1, RPB2, DED1, MRN1, NEW1	1.16E−06	13.30	2.40	mRNA binding (F)	3729
SSA1, SSA4, SSZ1, CCT8, KAR2, SSA2, HSP104, CPR6, HSC82, SSB2, HSP82	3.04E−06	8.60	0.90	Unfolded protein binding (F)	51,082
ILS1, CHA1, KRS1, HOM2, YDR341C, ARO10, SAM2, MET6, LEU1, TRP5, GUS1, VAS1, ADE3, MES1, YHR020W, THS1, URA2, CPA2, MET5, DPS1, AAT2, PDC1, ILV5, ILV2, LEU4, ADH1, IDH2, CDC60, GLN1, ASN1	4.47E−16	23.40	3.10	Cellular amino acid metabolic process (P)	6520
SSA1, SSZ1, SCP160, SSA2, NAM7, MKT1, SSB2, SSE1, NEW1	5.93E−07	7.00	0.50	Polysome (C)	5844
CCR4, DHH1, SUP35, PAB1, XRN1, TIF4631, YEF3, NAB6, MKT1, PRT1, MRN1	4.60E−06	8.60	1.00	Ribonucleoprotein granule (C)	35,770
COP1, SEC26, RET2, SEC27, SEC21	1.42E−05	3.90	0.10	COPI vesicle coat (C)	30,126
DHH1, SUP35, PAB1, XRN1, TIF4631, YEF3, NAB6, PRT1	6.27E−05	6.20	0.60	Cytoplasmic stress granule (C)	10,494

Additional protein groups were found, including protein folding factors and cellular metabolic processes. These proteins might be a result of experimental background, however some might represent a true RNA-associated proteins as recent papers show newly discovered RNA binding capabilities and suggest dual role for many proteins [2, 4, 27]. Experimental evidence [2, 3, 27, 28] for RNA binding is available in the literature for 62 of the 134 proteins identified herein (Additional file 1: Table S1).

In order to better distinguish between proteins associated with *PMP1* mRNA and those attached to streptavidin column or the MS2-CP-GFP-SBP fusion, another RaPID experiment was conducted with *PMP1*-*MS2L* mRNA and control *PMP1* mRNA without MS2 loops (Fig. 2c). Several bands are visible in the eluted material of *PMP1*-*MS2L* (+MS2L) which are absent in the control (−MS2L). The MS2-CP-GFP-SBP fusion protein is in equal amount in both lanes and serves as loading control. Five enriched bands in the *PMP1*-*MS2L* lane were cut and analyzed by mass spectrometry. Proteins identified are highlighted by boxes and indicated to the right. Peptides for five proteins were detected: Yef3p, Ssa1p, Tdh2 and Sam2p (which were detected also in the experiment presented in Fig. 2a) and Arc1p. Herein we focus on the elongation factor Yef3, as its high abundance, far above any other translation factor or ribosomes [29, 30], suggested roles beyond translation elongation.

### YEF3 interaction with *PMP1* mRNA

*YEF3* is an essential elongation factor which is unique to fungi. It interacts with the ribosome and eEFA1 and facilitates the release of tRNA during translation cycle [31–33]. Some elongation factors were shown to have a role in pathways other than protein translation, for example in nuclear export, apoptosis and proteolysis [34, 35]. Moreover, some were found to bind mRNA localization sites in the 3′ UTR of specific mRNAs [17, 36]. Yef3 protein contains three domains that enable its binding to rRNA [32, 37], but a direct binding to mRNAs was never demonstrated. We found that Yef3p is associated with *PMP1* mRNA in two independent RaPID experiments (Table 1; Fig. 2c). To further confirm these results, we performed western blot analysis with specific antibody against Yef3p (kindly provided by Prof. TG. Kinzy) (Fig. 2d). Equal amount of Yef3p are present in both input fractions of *PMP1* without and with MS2 loops (−MS2L and +MS2L respectively) yet observed only in the elution fraction of *PMP1*-*MS2L* (+MS2L). These results therefore confirm the isolation of Yef3p by tagged *PMP1* mRNA as was indicated by mass spectrometry analysis.

**Fig. 3:**
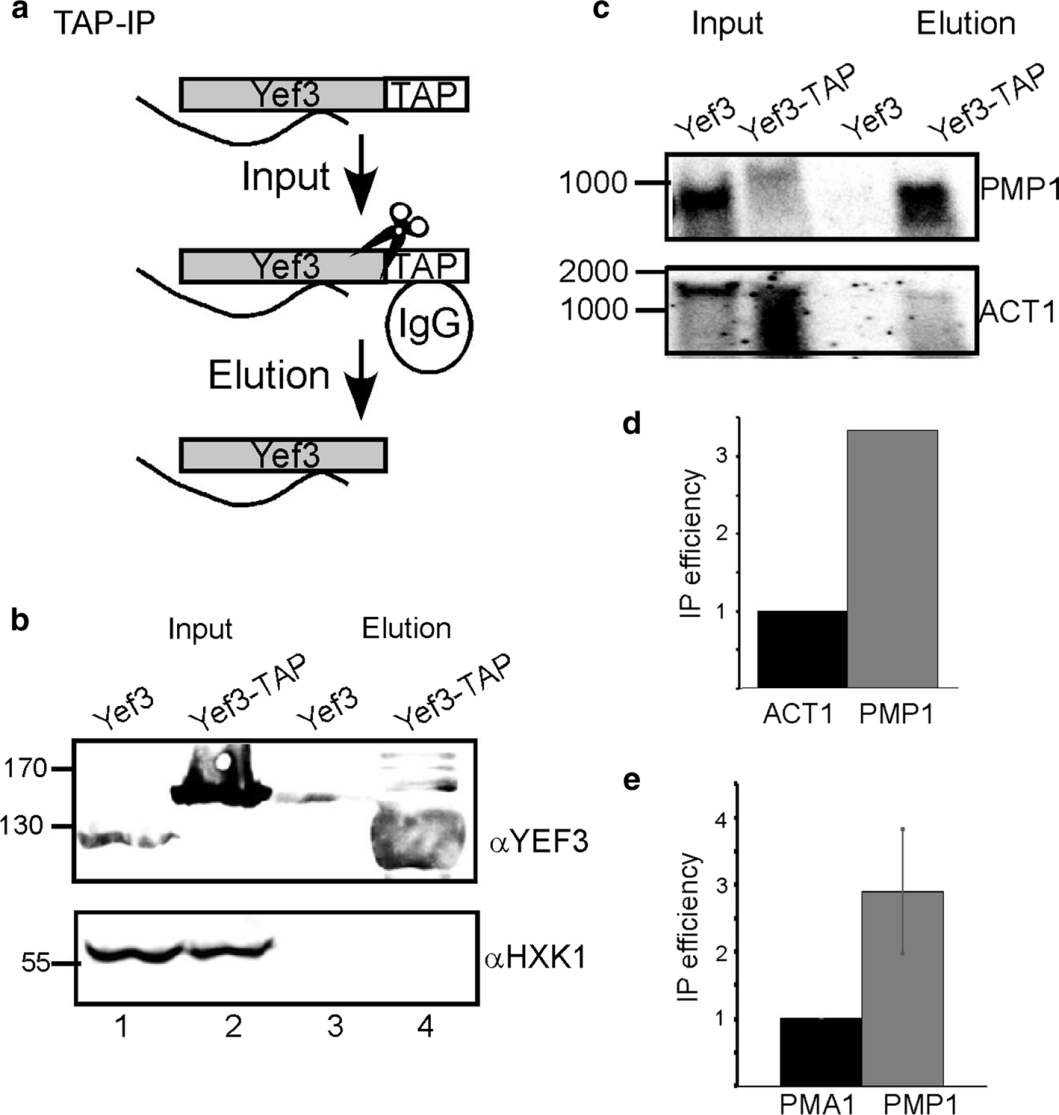
Validation of RaPID results by TAP purification. **a** TAP protocol. Cells expressing TAP-tagged Yef3 are lysed and loaded on IgG-Sepharose beads (*input*). The beads are washed extensively from non-specific binders and Yef3 is specifically cleaved off the beads by a TEV protease (depicted as scissors). Eluted material is analyzed for proteins (*panel* **b**) or mRNAs (*panels* **c**–**e**). **b** Western analysis with the indicated antibodies for aliquots from the Input and Elution. Note that the TAP tagged Yef3 is larger by ~25 kDa from the normal Yef3. Thus, the signal in *lane 3* is due to spill over from *lane 2* and not non-specific association of untagged Yef3. **c** Northern analysis for Input and Elution RNA samples isolated from a strain expressing untagged Yef3 or TAP-tagged Yef3. Membranes were hybridized with the probes indicated to the *right*. RNA size markers are indicated to the *left*. **d** Averages of two independent northern analyses. Results are presented as the ratio of Elution to Input samples, normalized to ACT1 efficiency. **e** RT-qPCR quantitation of the amounts of PMA1 and PMP1, isolated from the tagged (Yef3-TAP) strains. Results are presented as the ratio of Elution to Input, normalized to the PMA1 results. Results are from at least 3 independent biological repeats, from cell growth through IP to RT-qPCR analysis. *Error bars* are SEM of three independent biological repeats

Next, we wished to establish Yef3p interaction with the native *PMP1* mRNA. For that purpose co-IP experiment was performed with TAP tagged-Yef3 protein (*MAT*a *his3*Δ*1leu2*Δ*0met15*Δ*0ura3*Δ*0* YLR249w-Tap *HIS3 MX6*) (Fig. 3a). Cells expressing TAP-tagged Yef3 were harvested and incubated with IgG Sepharose beads recognizing the TAP moiety. Beads were then washed thoroughly and proteins were eluted using TEV protease. As control, the experiment was repeated with a strain in which Yef3p is untagged (Yef3). Western analysis revealed a highly specific and efficient purification of Yef3-TAP (Fig. 3b). RNA was extracted from the eluted material and subjected to northern analysis (Fig. 3c). While higher amounts of *PMP1* mRNA are apparent in the Input of Yef3 cells, and despite some RNA degradation, *PMP1* mRNA is detected only in the Elution fraction of the TAP-tagged Yef3p. This confirms that Yef3p binds *PMP1* mRNA. When the membrane was hybridized with a control probe (ACT1), a much lower association was apparent (Fig. 3c). This lower IP efficiency was apparent in two independent northern analyses (Fig. 3d). A quantitative analysis by RT-qPCR for another control mRNA (PMA1) revealed a threefold lower association (Fig. 3e).

### Yef3 interacts with mRNA in a ribosome-independent manner

**Fig. 4:**
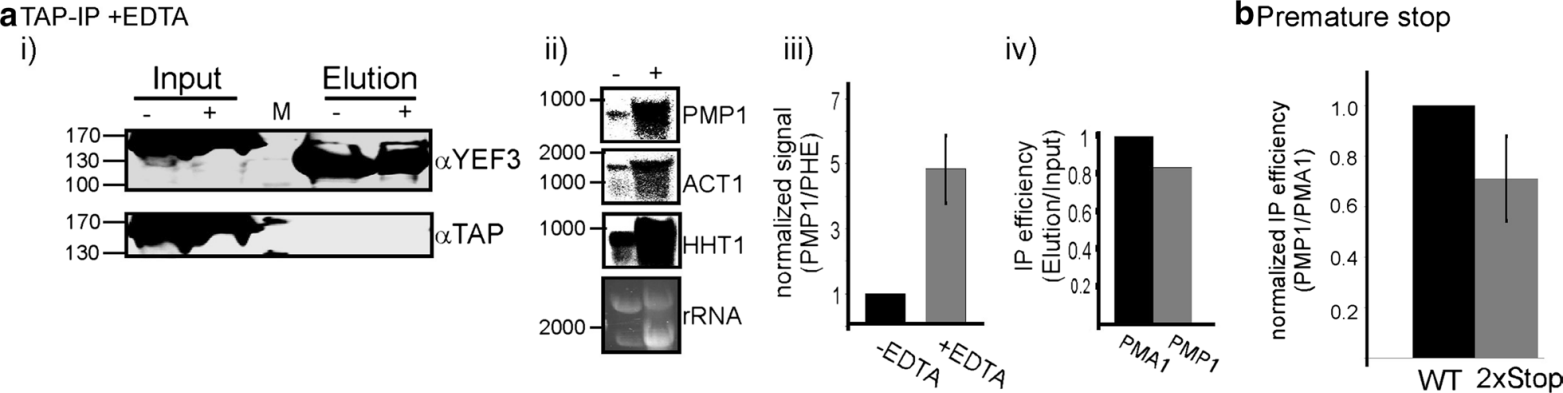
Yef3 is associated with mRNA in a ribosome-independent manner. Cells expressing TAP-tagged Yef3 were subjected to TAP purification and Yef3 associated mRNAs were analyzed. **a** Cells were lysed either in the absence (−) or presence (+) of 20 mM EDTA. (*i*) Protein samples from the input or the elution were subjected to western analysis with the indicated antibodies. The shorter bands detected in the Elution samples when αYef3 is used are consistent with cleavage of the TAP moiety by the TEV. Similarly, no signal is detected in the Elution when αTAP is used. (*ii*) Northern analyses with the indicated probes for RNA samples in the Elution samples from the minus EDTA (−) or plus EDTA (+) preparations. (*iii*) Quantitation of PMP1 isolated with Yef3-TAP by RT-qPCR. Samples were prepared either in the absence of EDTA (−EDTA) or its presence (+EDTA). To account for technical differences, an in vitro transcribed bacterial RNA (PHE, [51]) was spiked into each sample and its signals were used to normalize the PMP1 signals. (*iv*) RT-qPCR of PMA1 and PMP1, isolated in the presence of EDTA. **b** Yef3-TAP cells co-expressing either normal PMP1 (WT) or PMP1 with two stop codons immediately after the start codon (*2*× *Stop*) were subjected to TAP purification. RNA samples were subjected to RT-qPCR using primers for PMA1 and PMP1, and IP efficiency (Elution/Input) was determined. Values are normalized to the PMA1 signals

The results above indicate that Yef3 interacts with mRNAs. Yef3 is known to interact with translating ribosomes, and thus our observations might represent an indirect interaction that is mediated by the ribosome. Nevertheless, in our RaPID experiments hardly any ribosomal proteins were detected. Furthermore, although the ORF of PMA1 and ACT1 are much longer than the one of PMP1, their IP efficiency is much lower. This led us to suspect that Yef3 might interact with mRNA in a ribosome-independent manner. To experimentally explore this possibility, we performed lysis and purification of Yef3-TAP in a buffer containing 20 mM EDTA. This buffer induces complete polysomes disassembly, as revealed by analysis of polysomes sedimentation in sucrose gradient (data not shown). Interestingly, TAP purification in the presence of EDTA did not reduce the association of PMP1 with Yef3, but rather led to a much higher PMP1 signals than in the absence of EDTA (Fig. 4a ii). Higher amount in the presence of EDTA was apparent also for few other mRNAs (ACT1, HHT1 and PMA1). Quantitative analysis by RT-qPCR revealed a fivefold increase in PMP1 association, and slightly higher for PMA1 (Fig. 4a iii, iv). This may indicate that in the presence of EDTA the mRNA is more accessible for interaction with Yef3.

The results in the presence of EDTA strongly suggest that Yef3 interacts with mRNAs in a ribosome-independent manner. Yet, the presence of rRNA in the eluted fractions (Fig. 4a ii) raises the concern that some ribosomes are still associated with the mRNAs, even in the presence of EDTA. Furthermore, as the EDTA is added to the cell lysate, these interactions may occur post-lysis. Thus, to further exclude this possibility, we compared the association of Yef3-TAP with either normal PMP1 or PMP1 that contains two stop codons immediately downstream to the initiation codon. Cells were grown to logarithmic phase under optimal conditions (i.e. conditions in which PMP1 is translated) and subjected to TAP purification. Quantitative RT-qPCR revealed that the efficiency of precipitation of the 2xstop transcript is high and about 70 % of the one observed for the normal transcript (Fig. 4b). This indicates that excluding translation has negligible effect on association of Yef3 with PMP1 mRNA.

### Yef3 interacts with 3′ UTRs

**Fig. 5:**
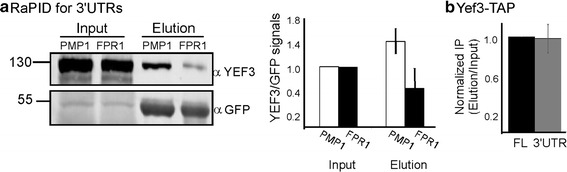
Yef3 interacts with 3′UTRs. **a** The 3′ UTR of either PMP1 or FPR1 were cloned downstream to multiple MS2 loops and introduced into cells expressing the MS2 fusion protein. Cells were subjected to RaPID and protein samples from the Input or the Elution were subjected to western analysis with the indicated antibodies. The histogram presents the normalized Yef3 signals in each fraction. *Error bars* represent SEM of three biological replicates. **b** Plasmids expressing either the full length PMP1 transcript (FL) or only the 3′ UTR were introduced into cells deleted of PMP1 and expressing TAP-tagged Yef3. Cells were subjected to TAP purification in the presence of EDTA (in order to eliminate any sporadic translation along the 3′ UTR), and the amount of the FL or the 3′ UTR was quantified by RT-qPCR. *Error bars* represent SEM of three biological repeats. Elution efficiency in each repeat was normalized to the efficiency of a control mRNA (PMA1)

To substantiate that Yef3 association can occur with untranslated RNAs in vivo, we constructed vectors in which the 3′ UTRs of either PMP1 or FPR1 were cloned downstream to MS2L (designated MS2L-PMP1 or MS2L-FPR1, respectively). These clones were introduced into yeast expressing the MS2-CP-GFP-SBP fusion protein, and subjected to RaPID analysis. Western analysis revealed that Yef3 co-purifies efficiently with both 3′ UTRs, with stronger efficiency to PMP1 3′ UTR (Fig. 5a).

We also performed the reciprocal experiment, utilizing TAP-tagged Yef3. Yef3-TAP was expressed in cells together with either PMP1 full length (FL) or only the 3′ UTR of PMP1. These cells were deleted of the endogenous PMP1, in order to eliminate chances of cross-signals. To minimize chances of fortuitous translation on the 3′ UTR, the TAP procedure was performed in the presence of EDTA, as in Fig. 4a. RT-qPCR for the Yef3 associated material revealed a significant association of the 3′UTR with Yef3-TAP, to the same level as the full length, ORF-containing transcript (Fig. 5b). These results further establish that Yef3 interacts with 3′ UTR.

## Discussion

We performed RaPID analysis in a search for proteins that bind PMP1, and may thus be involved in its localization. Proteomics studies thus far have utilized an oligo-dT isolation step, and thus identified proteins that are associated with the entire pool of mRNAs [4, 38]. Thus, to the best of our knowledge, this is the first study in which the repertoire of proteins that are associated with a particular mRNA were identified. We obtained a large number of proteins that were not known to bind PMP1. Among these a clear enrichment for RNA binding proteins, which further confirms the approach. In addition to the known RNA binding proteins, many of our positive hits were previously reported to bind RNA (Suppl. Table 1). Of note are various metabolic enzymes, including amino acids metabolism (15 % of our hits), energy metabolism (11 %) and fatty acid metabolism (4 %). A dual role for metabolic enzymes was recently reported, and may expose a hidden layer of their activity [2–4].

We note that most of the proteins that were detected by the RaPID analysis (Additional file 1: Table S1) are highly abundant ones. This is likely to be due to technical limitations, either insufficient purification or mass spectroscopy preference. Thus, the complete list of 3′ UTR binding proteins is probably much larger. Even though PMP1 3′ UTR is one of the longest in the yeast genome, and hence likely to bind more proteins than average, it still provides a glimpse to the extent of RNA–protein interactions. Many proteins are likely to be bound to each mRNA at any given time, and coordinate its function.

Yef3p (eEF3) is a ribosome-dependent ATPase that is essential for translation elongation in fungi. It interacts with eEF1A for insertion of aminoacyl-tRNA to the ribosome A-site, and facilitates the excision of deacyl-tRNA from the E-site [31–33]. To exert its function Yef3p has three domains that interact with the ribosome, a HEAT domain at the N-terminal, a middle chromodomain and a basic C-terminal domain [32]. Herein we present a novel interaction for Yef3, with non-coding regions of the mRNA. Analysis of protein levels reveals that Yef3 is highly abundant (870,000 copies per cell [29]), far above other translation factors (e.g. 1000× higher than eEF1A) and more than fourfold higher than the number of ribosomes in a yeast cell [30]. This significant excess coincides with roles outside of translation elongation. eEF3 was shown to directly interact with polynucleotides and rRNA [32, 37, 39] (Fig. 4a ii). Considering the interaction with rRNA, and the interaction with untranslated regions that is shown herein, it is not unlikely that eEF3 interacts also with other RNAs, such as tRNA or small RNAs. A credible and thorough analysis of this possibility is yet to be done.

The interaction of Yef3 with 3′ UTRs suggests regulatory roles that are implicated through this domain. Most common are effects on translation regulation, mRNA stability and mRNA localization [40–42]. Interestingly, such roles were previously shown to another elongation factor, eEF1A. eEF1A was found to bind sequences in the 3′ UTR of MT1 mRNA that are important for its perinuclear localization [17, 43]. eEF1A was also shown to bind the mRNAs of F-actin and β-actin and affect their cellular localization [19]. Recently, eEF1A was shown to bind the HSP70 mRNA, and affect its stability, transport and translation [21]. Interestingly, eEF1A (TEF1) appeared in our RaPID data (Table 1) to bind PMP1 mRNA and is known to physically interact with Yef3 [44]. This may suggest that these proteins may confer their post-transcriptional roles while in complex.

The data in Fig. 5b shows that the association of Yef3 with PMP1 3′ UTR is similar to its association with the full length transcript. The introduction of two stop codons at the beginning of the coding regions led to ~30 % decrease in association (Fig. 4b). Thus, most of Yef3 binding sites are within the 3′ UTR and are independent of ribosomes. It is unclear at this stage whether Yef3 has a specific binding motif, or can bind promiscuously to any site along the mRNA. Promiscuous binding is likely to correlate with the length of the mRNA. However, we do not see such correlation: PMA1 and ACT1 are longer than PMP1, yet in the presence of EDTA are bound less efficiently by Yef3 (Fig. 3d). Similarly, HHT1 is half the length of ACT1, yet bound as efficiently by Yef3 (Fig. 4a ii). This may suggest that Yef3 binds specific sites along the mRNA, and their number is unrelated to the length. Identifying the exact binding site of an RBP is a challenging task and may necessitate genome-wide methods, such as PAR-CLIP [45]. Yef3 appeared to be associated with all mRNAs that we tested herein (to varying extent), therefore we expect that its binding sites are present on many cellular mRNAs. Overall, in light of its high abundance, we speculate that Yef3 is a posttranscriptional regulator of many mRNAs.

## Conclusions

In this study we identified many novel proteins that interact with PMP1 mRNA. We thereby expand the knowledge of proteins that interact with mRNA. Importantly, our study allowed identification of proteins that are associated with a particular mRNA (i.e. PMP1), in contrast to previous global studies, that could not assign association with particular mRNA due to simultaneous isolation of a variety of mRNAs. Another significant finding herein is that the elongation factor Yef3 interacts with mRNA in non-coding regions and in a translation independent manner. These results suggest an additional, non-elongation function for this factor and thereby expose an unknown layer of activity for this factor.

## Methods

### Yeast growth conditions

Yeast strain containing PMP1-MS2L (*Mat* α, *his3∆1*, *leu2∆0*, *lys2∆0,ura3∆0* PMP1 ORF-MS2L-PMP13UTR (lab name yA553)) was made as described [26], on the genetic background of BY4742 (Mat α, *his3∆1*, *leu2∆0*, *lys2∆0,ura3∆0*). Yef3-TAP (*MAT*a *his3*Δ*1leu2*Δ*0met15*Δ*0ura3*Δ*0* YLR249w-Tap *HIS3 MX6* (lab name yA1128)) [29] is on the genetic background of BY4741 (Mat a, *his3∆1*, *leu2∆0*, *met15∆0*, *ura3∆0*) and was kindly provided by Dr. Ghil Jona, Weizmann Institute of Science. BY4741 and BY4742 were therefore used as parental controls were indicated. Plasmids used in this study are listed in Table 2. Cells were grown at 30 °C in YPD (1 % yeast extract, 1 % peptone, and 2 % dextrose) and plasmids were maintained by growing the cells in appropriate selection media (SD with the relevant supplementary).

**Table 2:** Plasmids used in this study

Lab name	Gene	Vector	Origin
pA262	PMP1 normal transcript	pRS415	[23]
pA263	PMP1 with 2 premature stop codons	pRS415	[23]
pA724	MS2-CP-GFP-SBP under MET promotor	puG36	[46]
pA872	12× MS2L-PMP1 3′ UTR under ADH1 promotor	pRS415	This study
pA873	12× MS2L-FPR1 3′ UTR under ADH1 promotor	pRS415	This study
pA922	PMP1 3′ UTR under ADH1 promotor	pRS415	This study

### Nucleic acids extraction and northern analysis

Genomic DNA and RNA were isolated by “Smash and Grab” and Hot phenol protocols, as described in [47, 48], respectively. For northern analysis RNA samples were separated by electrophoresis, blotted, and hybridized, essentially as described in [49]. Radiolabeled probes were prepared either from PCR product of the gene of interest labeled with α^32^P dCTP by random priming labeling kit (Biological Industries 20-101-25) or by labeling 5′-end DNA oligos with γ^32^P ATP by T4 polynucleotide kinase. Membranes were imaged with a Typhoon FLA 7000 PhosphorImager (GE healthcare) and signals were quantified with ImageQuant TL v7.0 software (GE healthcare).

### RNA binding proteins purification and identification (RaPID)

RaPID method was done essentially as in [25]. Briefly, Yeast cells (yA553 or its parental control) were grown to logarithmic phase (OD_600_ 0.8–1) in 500 ml of selective medium, washed with 1× PBS and transferred to 500 ml selective medium without methionine for 1 h to induce MS2-CP-GFP-SBP expression. Cells were incubated with 0.05 % formaldehyde in 1× PBS and slowly rotated at room temperature for 10 min to cross-link protein-RNA complexes. 0.125 M Glycine was added to stop the cross-linking reaction. Cells were then suspended in 5 ml cold RaPID buffer lysis (20 mM Tris–HCl pH 7.5, 150 mM NaCl, 0.5 % NP-40, 1.8 mM MgCl_2_, 1 mM DTT, 5 mg/ml Heparin, 1 mM PMSF, 10 μg/ml Pepstatin A, 10 μg/ml Aprotinin, 10 µg/ml Leupeptin, 10 µg/ml Soybean trypsin inhibitor) and cells were lysed in bead beater by two rounds of 90 s pulses. The lysate was then cleared by centrifugation at 10,000*g* for 10 min at 4 °C. Aliquot (1/50 and 1/100 of the lysate) were set aside for RNA and proteins samples respectively as “Input” sample. The lysate was then incubated with 300 μg Avidin (Sigma A9275) for 10 min at 4 °C to quench free biotin and biotinylated proteins and mixed with 250 μl streptavidin beads (GE Healthcare 17-5113-01) (pre-blocked with 4 % BSA and 0.1 mg of yeast tRNA (Sigma R8508). The lysate was incubated for 1 h at 4 °C in constant rolling (10 rpm) to allow binding of MS2-CP-GFP-SBP proteins to the streptavidin beads. The beads were then cleared by centrifugation (660 g, 2 min, 4 °C) and unbound material was washed three times with 1 ml RaPID lysis buffer, twice with 1 ml RaPID wash buffer (20 mM Tris–HCl pH 7.5, 300 mM NaCl, 0.5 % NP-40) and once with 1 ml 1xPBS. For elution of bound material, streptavidin beads were incubated with 120 μl of 6 mM biotin (Sigma B4501) in 1× PBS for 45 min. RNA was extracted from the eluted material by supplementing with equal volume of reverse crosslinking buffer (100 mM Tris–HCl pH 7.0, 10 mM EDTA, 20 mM DTT, 2 % SDS), heating at 65 °C for 45 min, and phenol extraction followed by ethanol precipitation. Proteins were extracted by adding equal volume of Laemmli buffer and heating at 65 °C for 45 min.

### Silver stain and mass spectrometry analysis

Proteins obtained from RaPID experiments were resolved on 10 % SDS PAGE and the gel was silver stained (Silver Stain Plus Kit, Bio-Rad 161-0449). Regions of interest were cut from the gel, proteins were extracted, digested by trypsin protease and peptides analyzed by LC–MS/MS on the OrbitrapXL mass spectrometer in Smoler Proteomics Center, Technion—Israel Institute of Technology. Data was analyzed using Discoverer software version 1.4 against the yeast section of the Uniprot database and against decoy databases (in order to determine the false discovery rate (FDR)), using the Sequest and the Mascot search engines. Only proteins with at least two identified unique peptides and FDR <0.01 are shown in Additional file 1: Table S1.

### Purification of Yef3- TAP associated mRNAs

Purification of Yef3-TAP with its associated mRNAs was done essentially as described in [50]. Briefly, 2 liters of TAP-tagged Yef3 (yA1128) or its parental control were grown to logarithmic phase (OD_600_ 0.8–1), washed two times with 40 ml of TAP-buffer A (20 mM Tris–Hcl pH 8, 140 mM KCl, 1.8 mM MgCl_2_, 0.1 % NP-40, 0.02 mg/ml Heparin), suspended with 5 ml of TAP-buffer B (TAP-Buffer A supplemented with 0.5 mM DTT, 1 mM PMSF, 0.5 µg/ml Leupeptin, 0.8 µg/ml Pepstatin, 0.2 mg/ml Heparin, 100 U/ml RNasin, 20 U/ml DNase1), and lysed in a bead beater. The lysate was cleared from cell’s debris by centrifugation at 10,000*g* for 10 min at 4 °C and 1/10 of the lysate was set aside for unprecipitated control (“Input”). Immunoprecipitation was performed by incubating the lysate with 400 µl of IgG beads, (pre-equilibrated with TAP-buffer A) for 2 h at 4 °C with constant rolling (10 rpm). Bound material was subjected to four rounds of washing with 4 ml ice-cold TAP-buffer C (20 mM Tris–Hcl pH 8, 140 mM KCl, 1.8 mM MgCl_2_, 0.01 % NP-40, 0.02 mg/ml Heparin, 0.5 mM DTT, 10 U/ml RNasin). Immunoprecipitated (IP) Proteins and RNA were eluted by TEV-protease (Invitrogen 12575-015) cleavage for 2 h at 16 °C in a TEV reaction buffer (provided by the manufacturer). The eluted RNA, together with the Input control, was supplemented with 20 mM EDTA to improve overall RNA extraction and then subjected to northern blot or RT-qPCR analysis.

### Reverse transcriptase quantitative PCR (RT-qPCR)

Input and elution RNA samples were spiked with 1 ng of in vitro transcribed PHE RNA, as control for all subsequent steps. Next, RNA was extracted with Trizol reagent (Ambion 15596-026) and heparin leftovers were removed by precipitation in 2 M LiCl, incubated at −20 °C for at least 30 min, and centrifuged at 16,000×*g* for 20 min at 4 °C, followed by standard ethanol-sodium acetate precipitation. RNA purity was tested by Epoch Spectrophotometer, and samples were then treated with DNase I (Ambion AM1907) and reverse transcribed with RT Supermix (Bio Rad #1708841) according to the manufacturer’s instructions. No-RT controls were made for each sample to test for DNA contamination. qPCR analysis was made with Power SYBR Green PCR Master Mix (Ambion 4367659) in 25 µl reaction volume, performed in Applied Bioscience Real-Time PCR 7300 System, and analyzed by Applied Bioscience 7500 software. Standard curves of Input and of Elution fractions were produced for efficiency calculation of the qPCR. Melting curves were used to verify that the PCR produced unique product. qPCR primers used are: Phe (F27) CCGTGAAGATGTATTGCCCG; Phe (R130) CGACCTTTTGAACGGCATCT, PMP1 3′ UTR (F154)TTCGTCCGTTCGCTTTACTG; PMP1 3′ UTR (R290) ACGGGATCTGTCTAAATACGAAAC, PMA1 (F2438) CTGGTCCATTCTGGTCTTCTATC; PMA1 (R2541) TCAGACCACCAACCGAATAAG.

## Additional file

10.1186/s12867-015-0045-5 Proteins identified in the mass spectroscopy analysis. List of 134 genes for which at least two identified unique peptides and FDR<0.01 were detected in the LC-MS/MS analysis. The table includes columns with the gene name, protein molecular weight, the number of peptides that were identified and whether there is experimental evidence for it to bind RNA.

## Abbreviations

RBP: RNA binding protein
RaPID: RBP purification and identification
eEF: eukaryotic elongation factor
UTR: untranslated region
TAP: tandem affinity purification
MS2L: MS2 phage RNA loop structure
MS2-CP: MS2 coat protein
SBP: streptavidin binding domain
LC-MS/MS: liquid chromatography tandem mass spectrometry
