# Supplementary material for: The elongation factor eEF3 (Yef3) interacts with mRNA in a translation independent manner
Source: BMC Mol Biol. 2015 Sep 24;16:17. doi: 10.1186/s12867-015-0045-5 (PMC4582935; doi:10.1186/s12867-015-0045-5)
Supplement: Supplementary file 1 — 10.1186/s12867-015-0045-5 Proteins identified in the mass spectroscopy analysis. List of 134 genes for which at least two identified unique peptides and FDR<0.01 were detected in the LC-MS/MS analysis. The table includes columns with the gene name, protein molecular weight, the number of peptides that were identified and whether there is experimental evidence for it to bind RNA. [file 12867_2015_45_MOESM1_ESM.docx]

**Supplementary Table 1: Proteins identified in the mass spectroscopy analysis**. All proteins with at least two identified unique peptides and FDR<0.01 are shown. For each protein the following information is presented: YORF – systematic name; Standard name; Mw – Calculated molecular weight in kDa. The number in bracket represents the area in Fig. 2A that was cut from the gel; Peptide – Number of unique peptides identified for the protein. Superscript denote that the protein was previously shown to bind RNA as follows: ^1^ Tsvetanova *et al* 2010; ^2^Scherrer *et. al* 2010; ^3^Klass *et. al* 2013; ^4^ Casolari *et. al* 2012, ^5^Saccharomyces Genome Database (SGD).

| **YORF** | **Standard Name** | **MW** | **Peptide** | **Functional Group** |
| --- | --- | --- | --- | --- |
| YNR016C | ACC1 | 250 (1) | 11 | Fatty acid metabolism |
| YJL130C | URA2 | 244 (1) | 24 | Amino-acid metabolism |
| YCR093W | CDC39 | 240 (1) | 5 | Transcription |
| YKL182W | FAS1 | 228 (1) | 6 | Fatty acid metabolism |
| YPL231W | FAS2 | 207 (1) | 3 | Fatty acid metabolism |
| YBR208C | DUR1 | 201 (1) | 13 | Others |
| YDL140C | RPO21 | 191 (1) | 8 | Transcription |
| YML103C | NUP188 | 188 (1) | 2 | Others |
| YGL206C | CHC1 | 187 (1) | 10 | Vesicle-mediated transport |
| YOR341W | RPA190^3^ | 186 (1) | 5 | Transcription |
| YOR326W | MYO2^4^ | 180 (1) | 6 | Motor proteins |
| YGL173C | XRN1^5^ | 175 (1) | 2 | mRNA binding proteins |
| YDR127W | ARO1 | 174 (1) | 8 | Amino-acid metabolism |
| YJR137C | MET5 | 161 (1) | 7 | Amino-acid metabolism |
| YMR012W | CLU1^5^ | 145 (1) | 6 | Translation |
| YKL215C | OXP1 | 140 (1) | 3 | Amino-acid metabolism |
| YOR151C | RPB2 ^3^ | 138 (1) | 8 | Transcription |
| YMR109W | MYO5 | 137 (1) | 3 | Motor proteins |
| YDL145C | COP1 | 135 (1) | 11 | Vesicle-mediated transport |
| YPR010C | RPA135 | 135 (1) | 6 | Transcription |
| YPL226W | NEW1^5^ | 134 (1) | 5 | Ribosome biogenesis |
| YJL080C | SCP160^5^ | 134 (1) | 4 | mRNA binding proteins |
| YOR086C | TCB1 | 133 (1) | 3 | Membrane proteins |
| YGL062W | PYC1 | 130 (1) | 19 | Energy metabolism |
| YBR218C | PYC2 | 130 (1) | 7 | Energy metabolism |
| YML117W | NAB6^5^ | 126 (1) | 3 | mRNA binding proteins |
| *YGR094W* | VAS1^5^ | 125 (1) | 2 | tRNA synthetases |
| YPL160W | CDC60^5^ | 124 (1) | 4 | tRNA synthetases |
| YJR109C | CPA2 | 123 (1) | 11 | Amino-acid metabolism |
| YBL076C | ILS1^5^ | 122 (1) | 7 | tRNA synthetases |
| YLR249W | YEF3^5^ | 115 (1) | 25 | Translation |
| YFR030W | MET10 | 114 (1) | 5 | Amino-acid metabolism |
| YIL125W | KGD1 | 114 (1) | 4 | Energy metabolism |
| YGL201C | MCM6 | 113 (1) | 5 | DNA replication |
| YMR080C | NAM7^5^ | 109 (1) | 6 | mRNA binding proteins |
| YHR027C | RPN1^1^ | 109 (1) | 4 | Proteasome associated proteins |
| YGR240C | PFK1 | 108 (1) | 5 | Energy metabolism |
| YGR162W | TIF4631^5^ | 107 (1) | 2 | Translation |
| YEL032W | MCM3 | 107 (1) | 2 | DNA replication |
| YGR204W | ADE3 | 102 (1) | 7 | Amino-acid metabolism |
| YDR238C | SEC26^1^ | 109 (2) | 6 | Vesicle-mediated transport |
| YPR019W | MCM4 ^3^ | 105 (1) | 4 | DNA replication |
| YML111W | BUL2 | 104 (1) | 3 | Proteasome associated proteins |
| YNL287W | SEC21 | 104 (2) | 6 | Vesicle-mediated transport |
| YIL075C | RPN2^2^ | 104 (2) | 3 | Proteasome associated proteins |
| YMR205C | PFK2^2^ | 104 (2) | 8 | Energy metabolism |
| YNL313C | EMW1 | 102 (2) | 5 | Cell wall biosynthesis |
| YLL026W | HSP104 | 102 (2) | 8 | Heat shock proteins |
| YER070W | RNR1 | 99 (2) | 3 | Nucleotide metabolism |
| YGL137W | SEC27^1^ | 99 (2) | 5 | Vesicle-mediated transport |
| YGL008C | PMA1^1^ | 99 (2) | 5 | Membrane proteins |
| YNL085W | MKT1 | 94 (2) | 10 | Others |
| YAL021C | CCR4 | 94 (2) | 2 | Transcription |
| YOR133W | EFT1^5^ | 93 (2) | 8 | Translation |
| YOR361C | PRT1^5^ | 88 (2) | 4 | Translation |
| YCL030C | HIS4 | 87 (2) | 4 | Amino-acid metabolism |
| YLR274W | MCM5^2^ | 86 (2) | 5 | DNA replication |
| YGL234W | ADE5,7 | 86 (2) | 3 | Nucleotide metabolism |
| YLR304C | ACO1 | 85 (2) | 3 | Energy metabolism |
| YGL009C | LEU1 | 85 (2) | 2 | Amino-acid metabolism |
| YGR264C | MES1^5^ | 85 (2) | 12 | tRNA synthetases |
| YIL078W | THS1^5^ | 84 (2) | 5 | tRNA synthetases |
| YBR080C | SEC18 | 84 (2) | 8 | Vesicle-mediated transport |
| YPL240C | HSP82 | 81 (2) | 3 | Heat shock proteins |
| YKL104C | GFA1 | 80 (2) | 9 | Cell wall biosynthesis |
| YGL245W | GUS1^5^ | 80 (2) | 27 | tRNA synthetases |
| YMR186W | HSC82^1^ | 80 (2) | 20 | Heat shock proteins |
| YKR001C | VPS1^3^ | 79 (2) | 3 | Vesicle-mediated transport |
| YLR143W | DPH6 | 78 (2) | 2 | Others |
| YCR084C | TUP1 | 78 (2) | 3 | Transcription |
| YOR317W | FAA1^3^ | 78 (2) | 3 | Fatty acid metabolizm |
| YHR020W | YHR020W^5^ | 77 (2) | 5 | tRNA synthetases |
| YMR246W | FAA4 | 77 (2) | 4 | Fatty acid metabolizm |
| YPL106C | SSE1^3^ | 77 (2) | 6 | Heat shock proteins |
| YKL126W | YPK1 | 76 (2) | 4 | Others |
| YGL026C | TRP5 | 76 (2) | 7 | Amino-acid metabolism |
| YDR172W | SUP35^5^ | 76 (2) | 4 | Translation |
| YLR153C | ACS2 | 75 (2) | 4 | Energy metabolism |
| YPL093W | NOG1^5^ | 74 (2) | 2 | Ribosome biogenesis |
| YMR108W | ILV2^3^ | 74 (2) | 9 | Amino-acid metabolism |
| YJL034W | KAR2^1^ | 74 (2) | 2 | Heat shock proteins |
| YDR380W | ARO10 | 71 (2) | 5 | Amino-acid metabolism |
| YJR045C | SSC1 | 70 (2) | 11 | Heat shock proteins |
| YER103W | SSA4 | 70 (2) | 3 | Heat shock proteins |
| YAL005C | SSA1 | 69 (2) | 23 | Heat shock proteins |
| YLL024C | SSA2 | 69 (2) | 6 | Heat shock proteins |
| YDR341C | YDR341C^5^ | 69 (2) | 4 | tRNA synthetases |
| YPL184C | MRN1^5^ | 68 (2) | 8 | mRNA binding proteins |
| YNL104C | LEU4 | 68 (2) | 2 | Amino-acid metabolism |
| YER036C | ARB1^5^ | 68 (2) | 12 | Ribosome biogenesis |
| YDL185W | VMA1 | 68 (2) | 3 | Membrane proteins |
| YDR091C | RLI1^5^ | 68 (2) | 3 | Ribosome biogenesis |
| YDR037W | KRS1^5^ | 67 (2) | 4 | tRNA synthetases |
| YNL209W | SSB1 | 66 (2) | 7 | Heat shock proteins |
| YOR027W | STI1^2^ | 66 (2) | 3 | Heat shock proteins |
| YOR204W | DED1^5^ | 65 (2) | 5 | mRNA binding proteins |
| YBL039C | URA7^1^ | 65 (2) | 3 | Nucleotide metabolism |
| YDR143C | SAN1 | 65 (2) | 2 | Proteasome associated proteins |
| YER165W | PAB1^5^ | 64 (2) | 4 | mRNA binding proteins |
| YPR145W | ASN1 | 64 (2) | 2 | Amino-acid metabolism |
| YLL018C | DPS1^5^ | 63 (2) | 6 | tRNA synthetases |
| YLR044C | PDC1 | 61 (3) | 5 | Energy metabolism |
| YJL008C | CCT8^1^ | 61 (2) | 10 | Cytoskeleton proteins |
| YFR051C | RET2 | 60 (2) | 3 | Vesicle-mediated transport |
| YHR064C | SSZ1 | 58 (2) | 3 | Heat shock proteins |
| YDL160C | DHH1^5^ | 55 (2) | 3 | mRNA binding proteins |
| YAL038W | CDC19 | 54 (2) | 8 | Energy metabolism |
| YER091C | MET6 | 52 (2) | 6 | Amino-acid metabolism |
| YPR080W | TEF1^5^ | 50 (3) | 3 | Translation |
| YLR027C | AAT2^3^ | 46 (3) | 3 | tRNA synthetases |
| YGL048C | RPT6 ^3^ | 45 (3) | 4 | Proteasome associated proteins |
| YBR025C | OLA1 | 44 (3) | 4 | Proteasome associated proteins |
| YLR355C | ILV5 | 44 (3) | 3 | Amino-acid metabolism |
| YLL039C | UBI4^2^ | 43 (2) | 3 | Proteasome associated proteins |
| YLR216C | CPR6 ^3^ | 42 (3) | 7 | Others |
| YDR502C | SAM2 | 42 (3) | 4 | Amino-acid metabolism |
| YDL022W | GPD1 | 42 (3) | 2 | Energy metabolism |
| YPR035W | GLN1 | 41 (3) | 5 | Amino-acid metabolism |
| YFL039C | ACT1 | 41 (3) | 4 | cytoskeleton proteins |
| YDL055C | PSA1^1^ | 39 (3) | 5 | Cell wall biosynthesis |
| YOR136W | IDH2 | 39 (3) | 3 | Energy metabolism |
| YJR068W | RFC2^3^ | 39 (3) | 2 | DNA replication |
| YCL064C | CHA1 | 39 (3) | 2 | Amino-acid metabolism |
| YDR214W | AHA1 | 39 (3) | 3 | Heat shock proteins |
| YDR158W | HOM2 | 39 (3) | 2 | Amino-acid metabolism |
| YOL086C | ADH1 | 36 (3) | 5 | Energy metabolism |
| YJR009C | TDH2 | 36 (3) | 2 | Energy metabolism |
| YMR116C | ASC1^5^ | 35 (4) | 3 | Translation |
| YLR340W | RPP0^5^ | 34 (4) | 3 | Ribosomal proteins |
| YBL030C | PET9^2^ | 34 (4) | 2 | Energy metabolism |
| YEL021W | URA3 | 29 (4) | 4 | Nucleotide metabolism |
| YGR253C | PUP2 ^3^ | 28 (4) | 3 | Proteasome associated proteins |
| YHL033C | RPL8A^5^ | 28 (4) | 2 | Ribosomal proteins |
| YNL178W | RPS3^5^ | 26 (4) | 8 | Ribosomal proteins |
